# To the Editors of the Medical and Physical Journal

**Published:** 1806-02

**Authors:** Lodge Hall

**Affiliations:** Cork


### *To the Editors of the Medical and Physical Journal.*

GENTLEMEN,

THE engraver, in copying from the drawing annexed to my communication on the Screw Tourniquet, inserted in No. 82 of your useful miscellany, has represented the small screw intended to stop the large perpendicular one, as flatted on *top* and divided, instead of a *nut* flatted on the *sides*, as delineated.

If I had intended a screw-driver should be used, I would hardly have termed it a "*simple*," "*unembarrassing*" method of stopping the instrument.

I observed this error the moment I cast my eyes on the plate; but as the general intent could be perfectly understood from it, I forbore troubling you on so trifling a circumstance, and should still have remained silent on the subject, but for Mr. Simmons noticing it in your last number.

That gentleman may think, from reading what I have just written, that the "*coincidence*" is now complete between the tourniquet he recommended in the 8th volume of Medical Facts and Observations, and mine in No. 82, of the Medical and Physical Journal. But so far from this being the case, I can inform Mr. Simmons that he did not comprehend the improvement (or alteration) I have proposed, either when he wrote the paper alluded to, in Medical Facts and Observations, or the still later one in No. 83, of the Medical and Physical Journal; and if he will "*favor me*," by perusing my last communication, and viewing minutely the plate, he will there find that I have devised a method of shortening the *large* screw *one-half*, and increasing its power in the same ratio.*

Had I known of Mr. Simmons having proposed the little screw in dispute, I should not have ascribed it to Mr. Byrom.

Further, with regard to the engraving, I by no means intend that the tourniquet should be made so thick and clumsy as there represented, nor need the *large* screw be near so long; Dr. Millet having obliged me by drawing it in that manner, that the nature of the proposed alteration might be more correctly understood.

*Cork,*
*January* 14, 1806.

I am, &c.

LODGE HALL.

---

* With a screw ONE INCH in length, the tape can be shortened EIGHT INCHES.

